# Prevalence of Asthma amongst Schoolchildren in Jordan and Staff Readiness to Help

**DOI:** 10.3390/healthcare11020183

**Published:** 2023-01-06

**Authors:** Arwa Nour, Ahmad R. Alsayed, Iman Basheti

**Affiliations:** 1 Department of Clinical Pharmacy and Therapeutics, Applied Science Private University, Amman 11931, Jordan; 2 Faculty of Medicine and Health, School of Pharmacy, The University of Sydney, Camperdown, NSW 2006, Australia

**Keywords:** children, school, asthma, prevalence, Jordan

## Abstract

Assessing asthma prevalence and management in schools is crucial. Improving school policies may reduce asthma morbidity and mortality. This study aimed to determine the prevalence of asthma among schoolchildren in Amman, Jordan. Second, we evaluated Jordanian school staff on asthma first-aid knowledge and competence. This cross-sectional study was conducted over five months in 2019. The researcher visited primary schools (private and public), and the availability of proper first-aid tools and teachers’ knowledge were assessed. The participated schools included ten public schools with 100 participating teachers and ten private schools with 100 participating teachers. Less than 25% of all schools reported having an asthma first-aid kit, and 65% reported having medical reports for chronic diseases, including asthma. The mean number of students in the schools involved in the study was 455.31 ± 212.92, out of whom 10.38 ± 7.26 were asthmatic children. The prevalence of asthma was 2.38% among schoolchildren in Amman, Jordan. Schools were found to have insufficient medical reports for the asthma children, in addition to a lack of first aid kits. The asthma knowledge of teachers in schools was weak. There is a need for educators to develop more awareness. These findings shed light on important concerns that require immediate attention.

## 1. Introduction

Asthma is the most prevalent chronic childhood disease, and it causes noticeable morbidity and mortality rates in both children and adults [1]. Asthma symptoms are found in 14% of children worldwide [1]. Morbidity associated with asthma affects the stage of asthma that can be measured through disability and premature death [2]. The greatest asthma rates are in children between 10–14 years old [3]. While asthma mortality rates among developed countries have remained stable over the last decade, the rate among developing countries is still increasing [4].

There are many common factors associated with these tragic deaths. These include inadequate clinical management, missing follow-up after hospitalization, overuse of reliever medications, less use of preventer medications, lack of asthma action plan, and supervision by a specialist [5].

Uncontrolled asthma can cause several harmful issues the patient has to live with, such as poor quality of life, poor psychological health [6], and an increased number of absent days from school [6], and all that can result in poor academic performance [7]. Dealing with acute asthma exacerbations is a critical issue of asthma management [8]. In an ideal world, each asthmatic person would have a current asthma action plan that specifically states how to manage symptoms in that person [1]. The Australian Asthma Management Guidelines found that an acute exacerbation of asthma can be either mild/moderate, severe, or life-threatening. Moderate-to-severe asthma symptoms are the inability to complete sentences in one breath, noticeable chest tightness, and using intercostal neck muscles during breathing [9]. International guidelines recommend using a short-acting beta 2 agonist (SABA) bronchodilator with a spacer and calling the ambulance when these symptoms are found. This management is called asthma first aid [10]. It is important to know that asthma first aid is just the first of many steps in managing a child who has experienced a severe asthma exacerbation [10]. International Asthma Guidelines were reviewed to define the adequate treatment of an acute exacerbation of asthma in the absence of an asthma action plan, known as asthma first aid (AFA). Generic protocols are available in the international guideline Global Initiative for Asthma (GINA) [11] as well as United Kingdom guidelines [12] and guidelines from The United States [13]. Each of these guidelines includes similar asthma first aid directions. A SABA is recommended to be administered via a spacer device. Each protocol states that a SABA should be given within the first minute and that urgent medical attention should be provided. The protocols differ in the timing between doses, and the United States guidelines [13] are the least prescriptive.

Children spend a long time at their schools; therefore, it is essential to ensure that there are asthma management procedures and policies in the schools. These policies should include guidelines on dealing with children in the case of medical emergencies and the day-to-day management of students with specific healthcare needs and chronic diseases [14]. Having an Individual Health Care Plan (IHCP), a list of a child’s healthcare needs, is one of the most important parts of the policy requirements. All children with a diagnosis that could mean they need medical help or an emergency procedure at school must have an IHCP [15]. Improvement of policies conducted within the school field positively affects asthma outcomes. It is related to reducing school absences, enhancing academic outcomes, and decreasing morbidities because of asthma [16].

In Mexico, only l6% of school nurses were previously found to have oxygen, 45% had peak flow meters, 20% had nebulizers, and no school nurse had reliever stocked for emergencies [17]. In Jordan, proper asthma control among children is not enough. This may be associated with a decreased health-related quality of life, a low use of asthma medicines, and high rates of passive tobacco smoking [18]. Although data on asthma and its treatment are available, more research is needed in developing countries to reach a much greater understanding of the disease and develop more unified management strategies with the least risk and cost.

No previous studies have assessed the prevalence of asthma amongst primary schools in Jordan, nor the level of education of school staff on asthma first aid knowledge and skills. Amman is the capital city of Jordan, a developing country located in the Middle East. Schools in Amman can be divided into public (free of charge) and private (fees applied). The aim of this study was to determine the prevalence of asthma amongst schoolchildren in Amman, Jordan and to assess the staff’s level of knowledge.

## 2. Materials and Methods

### 2.1. Study Design and Clinical Setting

This cross-sectional study was conducted over five months, from April to August 2019, at primary schools in Amman. In this study, the researchers are clinical pharmacists. Ethics approval was issued from the Institutional Review Board (IRB) committee at the Jordanian Ministry of Education (7/13/2360). The researcher visited public and private schools in order to look at the availability of asthma first-aid kits in the schools, medical records for chronic illness such as asthma, awareness of caregivers in the school on how to deal with sudden asthma attacks, and the prevalence of asthma within children in Jordan.

Inclusion criteria for the children included being under the age of 16 years, children included in the school during the study period, and children with intermittent or persistent asthma as defined by the Global Initiative for Asthma guidelines [1]. All schools who agreed to participate signed the informed consent forms.

### 2.2. Study Protocol

The clinical pharmacist went to private and public schools, selected randomly from the list of schools found in Amman. Some schools welcomed the idea, while others refused to cooperate, providing no apparent reason. For the schools approved to participate, the researcher first met with the management and provided study information and their role at the school.

Additionally, for schools that accepted to be a part of the study, the clinical pharmacist went to the school’s physician to evaluate their level of awareness and readiness to deal with asthmatic students in case of a sudden asthma attack and the availability of proper first-aid tools.

Additionally, the clinical pharmacist checked if the school had a special record for asthmatic students regarding their general information and what medicines they were using. After meeting the management and the doctor, the clinical pharmacist met with the teachers and gave them a questionnaire to fill out. The questionnaire evaluated the teacher’s knowledge of asthma and how they would react in case a sudden asthma attack happens to a student.

### 2.3. Data Collection Tools

For the purpose of data documentation and evaluation, the following questionnaires were used by the researcher via an interview style:

#### 2.3.1. Prevalence of Asthma amongst Schools Children

The schools’ managements were asked to report the number of children in the school and how many of them were asthmatic to identify the prevalence of asthma amongst schools.

#### 2.3.2. Asthma First-Aid Kit Availability

The schools’ clinics in charge were asked if they have an asthma first-aid kit to use in case of asthma attack and if they know how to use it correctly to give the maximum effectiveness.

#### 2.3.3. Availability of Medical Records

The schools’ clinics in charge were asked if they have medical records for the children with a chronic illnesses such as asthma and if they know the medications that are taken by every one of these children.

#### 2.3.4. Asthma Knowledge Questionnaire

The schools’ teachers were asked to complete the self-reported knowledge questionnaire to assess their knowledge about asthma and how to deal with the children if they have sudden asthma symptoms.

The questionnaire consisted of fourteen (true or false) items regarding asthma knowledge, giving a score out of 14.

### 2.4. Statistical Analysis

The Statistical Package for the Social Science (SPSS) software version 21.0 was used to analyze the collected data (IBM Corp, 2012). Descriptive statistical analysis was used to describe sample characteristics, including the mean and standard deviation. Before analyzing the collected data, outliers, skewness, and missing data were checked and handled. All assumptions for each proposed statistical test were tested before the implementation of the assigned statistical tests.

## 3. Results

Public (*n* = 13) and private (*n* = 14) schools were visited randomly by the researcher. In order to enter the schools and interview the staff legally, a certified letter from the Ministry of Education was handed in upon recruitment. Some schools refused to participate in the study (public, *n* = 3; private = 4). No reasons were provided for declining study participation. Therefore, the total number of schools participating in the study was 10 private and 10 public schools.

### 3.1. Prevalence of Asthma amongst Schools Children

The average prevalence of asthma amongst schools children was 2.38%, with a minimum of 0.67% and a maximum of 5.61% (Table 1). No significant difference in the prevalence between public and private schools was found (*p* = 0.909).

### 3.2. Asthma First-Aid Kit Availability

As shown in Table 2, the study sample included 10 public schools with 100 participating teachers and 10 private schools with 100 participating teachers. No more than 25% of schools reported having an asthma first-aid kit, and 65% reported having medical reports for chronic diseases, including asthma. The mean number of students in the schools (*n* = 13) involved in the study was 455.31 ± 212.92, out of whom 10.38 ± 7.26 were asthmatic children.

### 3.3. Availability of Medical Records

In our study sample, there were four private and three public schools with no medical records for the children with chronic diseases and no information about asthmatic children at these schools.

### 3.4. Asthma Knowledge Questionnaire

The proportion of the study sample answering each of the questions correctly in the asthma first-aid knowledge questionnaire ranged from 35.5% to 68.5%. The mean number of questions answered correctly was 7.03 ± 1.87 out of 14 (Table 3).

## 4. Discussion

This is the first cross-sectional study that unveils the prevalence of asthma amongst school children in Jordan. It was found that the average prevalence of diagnosed chronic asthma amongst children was 2.38%. In addition, the study identified the availability of asthma first aid kits and medical reports of schools, showing that 75% of schools in Amman, Jordan, do not have an asthma first aid kit, and 35% do not have medical reports for their asthmatic children. Regarding the awareness of teachers about asthma first aid knowledge, only 50.2% were able to answer all of the questions addressing knowledge about asthma correctly.

The asthma first-aid kit can be lifesaving to children with asthma. No school should be empty from having such a kit. It was surprising to know through this study that public schools are not allowed to keep a first aid kit for the management of asthma in school. In this study, only 25% of schools reported having an asthma first-aid kit. Jordan is not alone when it comes to these negative results. In Mexico, only l6% of school nurses were previously found to have oxygen, 45% had peak flow meters, 20% had nebulizers, and no school nurse had reliever MDIs stocked for emergencies [17].

In this study, only 65% of the schools reported having medical reports for children with chronic diseases, including asthma. In Massachusetts, Knorr et al. [19] previously showed that in half of all schools, nurses reported that 90–100% of their students with asthma had documentation in the health record of a provider diagnosis of asthma and/or asthma medication orders. Approximately 25% of the nurses indicated that 75–85% of student health records contained a diagnosis, and the remaining 25% reported that less than 75% had this documentation. Things have moved on since then; a study conducted in Azerbaijan schools found that all schools included in that study had medical reports for their students [20].

Asthma first-aid knowledge in school staff showed that the average total correct score was 68.9% in New York City [21]. The mean score of correct responses in Spain was 16.0 ± 4.8 points out of 31 [22]. Similar findings were reported among Saudi Arabian school teachers, where the mean total knowledge score of the sample was 9.69 ± 4.00 (mean score out of 20 ± SD). Only 14% of the teachers were confident about their skill and competence in caring for a student with asthma or assisting a student during an asthma attack [23]. Our findings were parallel to the Turkish and American studies, revealing that the proportion of correct answers to the asthma first-aid knowledge questions in schools ranges between 35.5% and 68.5%. In general, the mean number of questions answered correctly by this study sample was 7.03 ± 1.87 out of 14, indicating that more work needs to be done.

In Jordan, the Ministry of Higher Education would benefit children with asthma greatly if they enforced a law where medical reports and asthma first-aid kits are compulsory in all schools across the country, including both public and private schools. Based on the results obtained, it would be attractive as a plan to propose procedures and guidelines to be implemented in schools to improve knowledge of asthma and first aid.

This study comes with certain limitations. It was only conducted in the capital of Jordan. Hence it may not be generalized to the rest of the areas in the country. Rural areas can have a different prevalence of asthma and different school results from that shown in the capital. The healthcare system is usually richer and more easily reached in Amman capital than in the rural areas. Another limitation is the relatively small sample size.

## 5. Conclusions

This is the first study to assess the prevalence of asthma among schoolchildren in Jordan. The prevalence of asthma was 2.38% amongst both genders. Schools were found to lack medical reports for the asthma children, in addition to first aid kits. The asthma knowledge of the teachers in schools was weak. An increase in awareness amongst parents and teachers is critical. These results highlight urgent issues that need to be addressed as quickly as possible.

Future studies should look at increasing teachers’ knowledge concerning their students’ chronic diseases, including asthma. Strategies to manage the availability of asthma first aid kit in schools is essential.

Based on the results obtained, it would be attractive to propose procedures and guidelines to be implemented in schools to improve knowledge of asthma and first aid.

## Figures and Tables

**Table 1:** Prevalence of asthma amongst schools children.

School	Asthmatic Students/All Students
Private school number 1, *n* (%)	27/840 (3.21)
Private school number 2, *n* (%)	5/430 (1.16)
Private school number 3, *n* (%)	4/600 (0.66)
Private school number 4, *n* (%)	NA
Private school number 5, *n* (%)	NA
Private school number 6, *n* (%)	16/720 (2.22)
Private school number 7, *n* (%)	NA
Private school number 8, *n* (%)	NA
Private school number 9, *n* (%)	22/550 (4.0)
Private school number 10, *n* (%)	10/360 (2.77)
Public school number 1, *n* (%)	4/195 (2.05)
Public school number 2, *n* (%)	6/107 (5.60)
Public school number 3, *n* (%)	4/286 (1.40)
Public school number 4, *n* (%)	NA
Public school number 5, *n* (%)	7/322 (2.17)
Public school number 6, *n* (%)	12/457 (2.62)
Public school number 7, *n* (%)	4/324 (1.23)
Public school number 8, *n* (%)	NA
Public school number 9, *n* (%)	14/728 (1.92)
Public school number 10, *n* (%)	NA
Average prevalence of asthma for all schools, (%) Average prevalence of asthma for public schools, (%) Average prevalence of asthma for private schools, (%)	2.38% Mean of percentages = 2.43 (1.48) Mean of percentages = 2.34 (1.26) *p* = 0.909

*n*: Number of patients. NA = Schools declined from informing the researcher the total number of students and the number of asthmatic students.

**Table 2:** Presence of asthma first-aid kit in schools.

Parameter	Total Number of Patients
Type of schools, *n* (%)	
Private	10 (50.0)
Public	10 (50.0)
Asthma first-aid kit, *n* (%)	
Yes	5 (25.0)
No	15 (75.0)
Asthma reports, *n* (%)	
Yes	13 (65.0)
No	7 (35.0)
Asthmatic students recorded, mean (SD)	10.38 (7.26)
All students recorded, mean (SD)	455.31 (212.92)

*n*: Number of patients; SD: Standard deviation.

**Table 3:** Asthma first-aid knowledge in schools.

Parameter	All Schools	Private Schools	Public Schools	*p* Value
1 Spacers are not recommended when giving asthma first aid to children (F), *n* (%)				0.322
Correct	97 (48.5)	52 (52.0)	45 (45.0)	
Incorrect	103 (51.5)	48 (48.0)	55 (55.0)	
2 An ambulance should still be called, when a child known to have asthma is able to breathe normally after reliever inhalers have been used (F), *n* (%)				**0.035**
Correct	134 (67.0)	74 (74.0)	60 (60.0)	
Incorrect	66 (33.0)	26 (26.0)	40 (40.0)	
3 When a person is having a sudden onset of asthma symptoms you should lie them on their side (F), *n* (%)				0.479
Correct	97 (48.5)	51 (51.0)	46 (46.0)	
Incorrect	103 (51.5)	49 (49.0)	54 (54.0)	
4 Wheeze, cough, and difficulty in breathing, are signs that a person needs to use reliever inhalers immediately (T), *n* (%)				0.447
Correct	137 (68.5)	66 (66.0)	71 (71.0)	
Incorrect	63 (31.5)	34 (34.0)	29 (29.0)	
5 Reliever inhalers are usually blue (T), *n* (%)				0.322
Correct	101 (50.5)	54 (54.0)	47 (47.0)	
Incorrect	99 (49.5)	46 (46.0)	53 (53.0)	
6 Administering high doses of reliever medicines to a child who is having a sudden onset of asthma symptoms is unlikely to cause harm (T), *n* (%)				0.883
Correct	71 (35.5)	35 (35.0)	36 (36.0)	
Incorrect	129 (64.5)	65 (65.0)	64 (64.0)	
7 When administering asthma first aid it is important to ask the child to hold their breath after each puff of reliever medicine is delivered via the spacer (F), *n* (%)				0.47
Correct	79 (39.5)	42 (42.0)	37 (37.0)	
Incorrect	121 (60.5)	58 (58.0)	63 (63.0)	
8 When administering asthma first aid you should not waste time by shaking the reliever inhaler (F), *n* (%)				0.184
Correct	71 (35.5)	40 (40.0)	31 (31.0)	
Incorrect	129 (64.5)	60 (60.0)	69 (69.0)	
9 Reliever inhalers only contain anti-inflammatory medicines (F), *n* (%)				0.318
Correct	113 (56.5)	60 (60.0)	53 (53.0)	
Incorrect	87 (43.5)	40 (40.0)	47 (47.0)	
10 When administering asthma first aid, it is best to put 4 puffs of reliever medicine into the spacer at one time and ask the person to take 4 big breaths (F), *n* (%)				0.258
Correct	104 (52.0)	48 (48.0)	56 (56.0)	
Incorrect	96 (48.0)	52 (52.0)	44 (44.0)	
11 It is possible for a person with mild asthma to have a severe asthma attack (T), *n* (%)				0.080
Correct	124 (62.0)	68 (68.0)	56 (56.0)	
Incorrect	76 (38.0)	32 (32.0)	44 (44.0)	
12 When a child has a sudden onset of asthma symptoms, they should drink lots of liquids such as water (F), *n* (%)				0.064
Correct	89 (44.5)	51 (51.0)	38 (38.0)	
Incorrect	111 (55.5)	49 (49.0)	62 (62.0)	
13 When asthma symptoms occur suddenly, taking inhaled corticosteroids will make the narrowed breathing tubes (airways) wider so it is easier to breathe (F), *n* (%)				**<0.001**
Correct	72 (36.0)	24 (24.0)	48 (48.0)	
Incorrect	128 (64.0)	76 (76.0)	52 (52.0)	
14 When administering asthma first aid, it is recommended to wait 4 min and assess the child after delivering the first 4 puffs of reliever medicine (T), *n* (%)				0.886
Correct	117 (58.5)	58 (58.0)	59 (59.0)	
Incorrect	83 (41.5)	42 (42.0)	41 (41.0)	
Number of correct answers for all the questions, mean (SD)	7.03 (1.87)	7.23 (1.74)	6.83 (1.99)	0.440

*n*: Number of patients; SD: Standard deviation.

## Data Availability

Not applicable.
